# Hooking injury, physiological status and short-term mortality of juvenile lemon sharks (*Negaprion bevirostris*) following catch-and-release recreational angling

**DOI:** 10.1093/conphys/cot036

**Published:** 2014-02-05

**Authors:** Andy J. Danylchuk, Cory D. Suski, John W. Mandelman, Karen J. Murchie, Christopher R. Haak, Annabelle M. L. Brooks, Steven J. Cooke

**Affiliations:** 1 Department of Environmental Conservation, University of Massachusetts Amherst, 160 Holdsworth Way, Amherst, MA 01003-9285, USA; 2 Department of Natural Resources and Environmental Sciences, University of Illinois at Urbana-Champaign, 1102 South Goodwin Avenue, Urbana, IL 61801, USA; 3 John H. Prescott Marine Laboratory, New England Aquarium, Central Wharf, Boston, MA 02110-3399, USA; 4 School of Chemistry, Environmental and Life Sciences, Department of Biology, College of The Bahamas, Freeport, Grand Bahama, Bahamas; 5 Cape Eleuthera Institute, Eleuthera, The Bahamas; 6 Fish Ecology and Conservation Physiology Laboratory, Department of Biology and Institute of Environmental Science, Carleton University, 1125 Colonel By Drive, Ottawa, Ontario, Canada K1S 5B6

**Keywords:** Catch and release, hooking injury, juvenile lemon shark, mortality, physiological stress, recreational angling

## Abstract

Sport fishing for sharks, including fishing with the intent to release, is becoming more prevalent within the recreational angling community. Common targets of recreational anglers are juvenile lemon sharks (*Negaprion brevirostris*) that frequent shallow tropical nearshore habitats. In this study, we captured 32 juvenile lemon sharks (530–875 mm total length) with conventional angling gear (i.e. spinning rods, dead fish bait and 5/0 barbed circle hooks) from the coastal waters of Eleuthera, The Bahamas, to determine the consequences of capture for individual sharks. Each shark was examined for hooking injuries, blood sampled to quantify physiological disturbance, assessed for reflex impairment and then monitored to assess post-release behaviour and mortality. Four sharks (12.5%) died following release during the 15 min tracking period. Principal components (PC) analysis revealed four axes describing 66.5% of the variance for blood physiology parameters, total length and water temperature. The PC1 and PC3 scores, characterized by positive factor loadings for indicators of exercise-induced stress and blood ion concentrations, respectively, were significantly related to fight time but were not associated with short-term mortality. Short-term mortality was significantly related to factor scores for PC4 that loaded heavily for water temperature and total length. Ten sharks (31%) exhibited impaired reflexes, with loss of bite reflex being most prevalent. Sharks that died had the following characteristics: (i) they had two or more impaired reflexes; (ii) they were hooked in the basihyal; (iii) they exhibited no movement after the initial bout of directional swimming; and (iv) they experienced high water temperatures (i.e. >31°C). Collectively, these results indicate that for juvenile lemon sharks inhabiting tropical flats, fight time can influence the degree of physiological disturbance, while water temperature contributes to the likelihood of survival following release.

## Abstract (precis)

Juvenile lemon sharks were angled and hooking injury, physiological stress, reflex impairment and short-term post-release mortality were quantified. Fight time was correlated with the level of physiological disturbance, but not post-release mortality. Four sharks (12.5%) died following release, and mortality was related to water temperature and body size.

## Introduction

Many shark populations worldwide are threatened by anthropogenic activities, including direct commercial harvest (Myers *et al.*, 2007) and incidental fishing capture (McKinnell and Seki, 1998; Lewison *et al.*, 2004; Molina and Cooke, 2012). Despite reported population declines (Worm *et al.*, 2013) and vulnerable life-history attributes (e.g. late age at maturity and low fecundity; Musick *et al.*, 2000), the targeting of sharks via hook-and-line angling as a form of recreation appears to be increasing (Skomal, 2007). Recreational angling is a common leisure activity worldwide (Cowx, 2002; Arlinghaus and Cooke, 2008), and catching sharks continues to be promoted and glamorized in the popular media (e.g. television, angling magazines and the Internet). Recreational shark fishing tournaments are popular (Holts *et al.*, 1998; Gallagher *et al.*, 2012), and sharks are also landed as bycatch when recreational anglers target other fish species (McPhee *et al.*, 2002; Dicken *et al.*, 2006).

As awareness of the ecological and economic importance of sharks grows, catch-and-release angling (rather than catch-and-kill angling) is being advocated or even mandated as a measure to help protect shark populations (reviewed by Skomal, 2007). Nevertheless, the conservation benefits of catch-and-release angling for sharks can only be achieved if the impacts on released individuals are minimized (Cooke and Suski, 2005). Despite an increasing body of work examining the physiological repercussions and mortality from commercial fishing capture and handling of sharks (e.g. Manire *et al.*, 2001; Morgan and Carlson, 2010; Brooks *et al.*, 2012; Frick *et al.*, 2012; Skomal and Mandelman, 2012 for a review), few studies to date have examined recreational hook-and-line capture in elasmobranchs. Among those that have, angling has been shown to inflict physical injury and pathology due to hook retention (Borucinska *et al.*, 2002) and to elicit metabolic disturbances (Hoffmayer and Parsons, 2001; Hoffmayer *et al.*, 2012), including those associated with the exercise/fight time (Skomal and Chase, 2002; Brill *et al.*, 2008; Heberer *et al.*, 2010). Overall, there is a need for better understanging of aspects of the angling and handling process, the resulting physical injury and physiological disturbance and the post-release survivorship in sharks to enable the development of best practices for catch-and-release recreational angling of sharks (Gallagher *et al.*, 2012).

Sharks with a nearshore distribution and high degree of site fidelity could be more accessible to recreational anglers and, in turn, more susceptible to angling-related impacts. One such species is the lemon shark (*Negaprion bevirostris*), found in the Atlantic Ocean from the USA to Brazil, in the waters of some West African countries and in the Pacific Ocean from Baja, California to Ecuador (Compagno, 1984). Neonates and small juvenile lemon sharks remain close to shore during the first few years of their life, where they tend to have a small home range (∼2–3 km^2^) and display a relatively high degree of site fidelity associated with tidal creeks and embayments (Morrissey and Gruber, 1993a,b; Murchie *et al.*, 2010). Direct capture, bycatch in commercial and recreational fisheries and the loss of critical nearshore habitats have contributed to the decline of lemon shark populations throughout their range (Manire and Gruber, 1990; Camhi *et al.*, 1998), prompting this species to be categorized as ‘near threatened’ on the IUCN Red List in 2009. Nevertheless, recreational anglers specifically seek out small lemon sharks on shallow tropical flats, either as a primary target or when the encounter rates of other nearshore target species, such as bonefish (*Albula* spp.) and permit (*Trachinotus falcatus*), are low (Kauffman, 2000). Even though many of these anglers voluntarily practise catch-and-release fishing, there may be disparities between the ways in which sharks are perceived and handled when compared with potentially less dangerous teleost fishes. Moreover, differences between the ways in which teleosts and elasmobranchs respond to angling-induced stressors may require specific guidelines for the effective catch and release of sharks (Cooke and Suski, 2005). Identification of the impacts associated with the recreational capture, handling and release of juvenile lemon sharks is vital for the development of best conservation practices for this species.

The purpose of our study was to evaluate hooking injury, physiological disturbance, post-release behaviour and short-term mortality of juvenile lemon sharks following catch-and-release recreational angling in The Bahamas. We expected that juvenile lemon sharks expressing greater signs of physical injury and physiological disturbance would be more prone to short-term mortality following release. We also anticipated that elevated physiological stress of juvenile lemon sharks would be associated with longer fight times, similar to what has been observed for sharks caught in commercial fishing gears and for teleost fishes captured via recreational angling.

## Methods

### Study site and capture methods

The study took place in between 23 and 27 June 2010, along the north coast of Cape Eleuthera, The Bahamas (25°54′N, 76°20′W). Previous work has shown that juvenile lemon sharks in these waters are relatively abundant (Danylchuk *et al.*, 2007a) and exhibit a high degree of site fidelity (Murchie *et al.*, 2010). This region of Eleuthera consists of small bays, tidal creeks and coastal flats. Sharks angled for this study were located in Page Creek, Kemp's Creek, Broad Creek, Starved Creek and the shoreline connecting these tidal creeks, including Poison Flat (see Figure 1 of Murchie *et al.*, 2013). The tidal creeks are lined with mangroves (*Rhizophora mengal* and *Avicennia germinans*), and the tidal range for the study area is ∼1 m.

**Figure 1.**
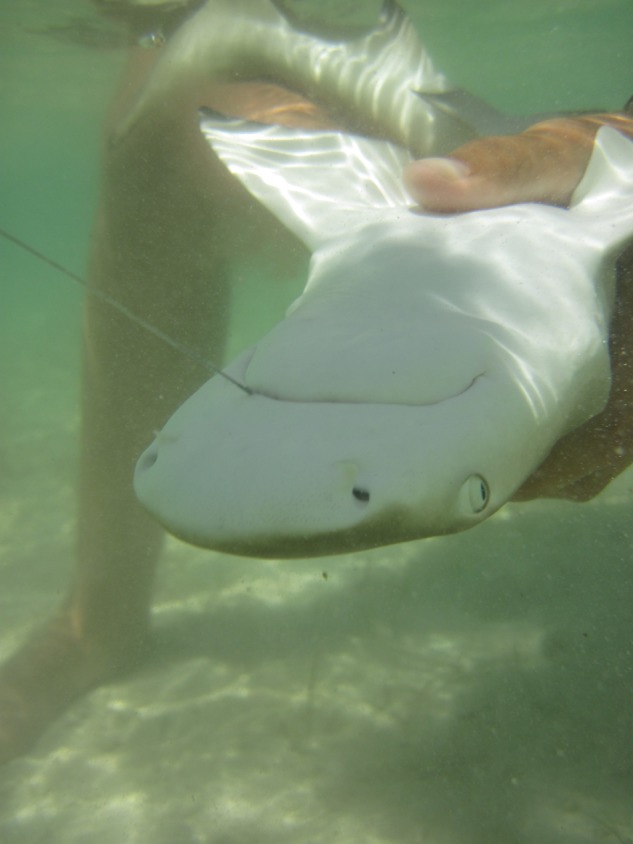
Juvenile lemon shark being held in tonic immobility prior to hook removal. Note that the hook is not visible.

Juvenile lemon sharks were visually located by anglers standing in a small boat or wading in the shallow water (<1.5 m deep). Conventional spinning tackle (1.8–2.1 m medium action rods and 4.5 kg test line) was used to cast a small piece of bait (Atlantic bonito, *Sarda sarda*) near the shark. Although likely to result in a more conservative estimate of hooking injury, we opted to use small barbed circle hooks (5/0) at the end of a wire leader (20–25 cm; 22–45 kg test) in an effort to reduce gut hooking and increase the probability of landing the shark (see review by Cooke *et al.*, 2012). If hooked, the angler fought the shark so that the duration of the angling event reflected the fighting capacity of the catch. All sharks were landed next to a small boat that then acted as a stable platform for all of the physical and physiological assessments. All procedures that followed were conducted with the mouth and gills of the shark being fully or partly submerged (i.e. we opted to eliminate air exposure as a potential impact).

### Quantification of the angling event and determination of shark characteristics

Fight time was measured from the time when the shark was hooked to the time when it was landed (to the nearest second). The total length of each shark was measured (to the nearest millimetre) and sex was determined by the presence/absence of claspers (Compagno, 1984). Sharks were examined for the presence of an umbilical scar as a means to differentiate neonates from older juveniles (Compagno, 1984). Following an assessment of hooking injury (see next subsection), an attempt was made to remove the hook without causing additional physical damage. If the hook was set in the oesophagus or deeper in the gut, the line was cut as close to the eye of the hook as possible. For other hooking locations (such as corner jaw), the hook was removed by using bolt cutters to cut the tip of the hook below the barb, followed by removal of both parts of the hook from the shark.

### Hooking injury

Hook placement was categorized as critical (gills, gullet or eye) or as non-critical (jaw, hinge, roof of mouth or basihyal) using similar criteria to those developed by Meka (2004) and Arlinghaus *et al.* (2008). We considered being hooked in the eye as a critical injury because it could greatly impair vision and, in turn, feeding or anti-predator behaviour. Hooking depth (measured from the tip of the snout to the point of hook entry) was measured and corrected for total length for comparison between sharks of different sizes (calculated as the proportion of hooking depth to total length of the shark; Cooke *et al.*, 2001; herein termed ‘length-corrected hooking depth’). The presence/absence of blood at the hooking site was noted, as was other overt physical damage related to angling (e.g. shark wrapped up in the leader).

### Physiological assessments

Once landed, juvenile lemon sharks were held in tonic immobility (Brooks *et al.*, 2011; Figure 1) in the shallow water while a non-lethal blood sample (1.5 ml) was obtained. A 3 ml vacutainer (lithium heparin coated; B-D Inc., Franklin Lakes, NJ, USA) and a 3.8 cm (1.5″) 21 gauge needle were used to draw blood from the caudal vasculature. The time to bleed was recorded based on the total cumulative time between the shark being hooked and the phlebotomy (to the nearest second). Blood samples were immediately placed in an ice-water slurry inside a cooler and processed within 15 min of collection.

Glucose and lactate levels were measured on whole blood by adding 10 μl of blood to hand-held glucose (ACCU-CHECK glucose meter; Roche Diagnostics, Basel, Switzerland) and lactate meters (Lactate Pro LT-1710 portable lactate analyzer; Arkray Inc., Kyoto, Japan; see Cooke *et al.*, 2008 for validation study with fish). A sample of whole blood was then spun using a micro-haematocrit centrifuge (ZIPocrit; LW Scientific, Lawrenceville, GA, USA) for 5 min at 4400***g*** to determine packed cell volume as the proportion of red cells to the total volume of the sample. The remainder of the whole blood was centrifuged (Clay Adams Compact II Centrifuge) for 5 min at 1163***g*** to separate red cells from plasma. Plasma was transferred to cryovial tubes and then frozen in liquid nitrogen for transport back to the laboratory and stored at −80°C prior to further analyses. Plasma sodium and potassium concentrations were measured using a flame photometer (Cole-Palmer Single-Channel Digital Flame Photometer, model WU-02655-00, Vernon Hills, IL, USA). Plasma chloride, magnesium, calcium, blood urea nitrogen (BUN) and creatinine were measured using commercially available kits (BioAssay Systems, Hayward, CA, USA; chloride DICL-250, magnesium DIMG-250, calcium DICA-500, BUN DIUR-500 and creatinine DICT-500).

### Reflex indices and post-release tracking

Following hook removal and phlebotomy, sharks were taken out of tonic immobility and tested for a series of reflexes as a cumulative assessment of vitality before release (Davis, 2010). Reflex indices have been used successfully in the past to predict post-release mortality in fishes (e.g. Davis, 2007; Campbell *et al.*, 2010; Raby *et al.*, 2012), although only in two studies to date with sharks (Braccini *et al.*, 2012; Gallagher *et al.*, In Press). We used a reflex impairment index modified from the previously developed reflex action mortality predictors (RAMP) method (Davis, 2007). Reflexes were assessed by the same team of individuals to standardize the procedure among sharks.

Each reflex was assessed categorically (0 = unimpaired; 1 = impaired) in a conservative matter; that is, if the team of handlers were doubtful about whether a reflex was present, it was recorded as being impaired. Tested reflexes included the following: bite response (BITE); ability to close the nictitating membrane over the eye (NM); flex of the body (FLEX); and equilibrium at release (EQUIL). BITE was noted as impaired if the shark did not bite down on a smooth metal rod inserted into its mouth that touched the entrance to the oesophagus. NM was observed by lightly touching the eye with a smooth-ended metal rod and scored as impaired if the nictitating membrane did not rapidly (i.e. within 2 s) cover the eye when responding to the stimuli. FLEX was impaired if the shark did not tense its muscles and attempt to struggle free when held by the tail immediately prior to release. Finally, EQUIL was noted as impaired if the shark was not able to stay upright and swim away following release by the researcher. From the reflex results for each fish, we calculated a RAMP score as a simple proportion of the four measured reflexes that were impaired in an individual fish (0 = no reflexes impaired; 1 = all reflexes impaired; Davis, 2007; Raby *et al.*, 2012).

Immediately prior to testing for EQUIL, a visual float was attached to each shark to facilitate the quantification of post-release behaviour. Visual floats consisted of a small hook tied to 3–4 m of 1.8 kg test monofilament line attached to a 3 cm brightly coloired, oval fishing bobber. The small hook was inserted just below the posterior origin of the first dorsal fin. Other species occupying the same habitat have previously been tracked post-release using visual floats (Cooke and Philipp, 2004). Once tagged, sharks were released within 5 m of their point of capture, and the water temperature was measured (to the nearest 0.1°C). All juvenile lemon sharks were visually tracked for 15 min post-release, and their locations during this time period were noted on a site map. Sharks were tracked for 15 min because of a combination of logistical constraints and that a 15–20 min window of time post-release was shown to be the most critical for short-term survival for another species in this environment (Danylchuk *et al.*, 2007b). Activity patterns recorded continuously during the monitoring period included swimming directionally, swimming non-directionally and not moving. Swimming directionally (SD) was defined as continuous forward motion in a well-defined track. Swimming non-directionally (SND) was defined as continuous forward motion but in circles or meandering within a relatively small area (∼5–10 m^2^). A shark was deemed not moving (NM) if its position was stationary for more than 5 s. A shark NM was deemed dead once we were able to approach the individual physically, to observe a lack of movement of the gill slits indicating no respiration for 5 min, and the body was limp when handled. In all cases, the visual floats were removed following the observation period by rapidly approaching the shark on foot or by boat and pulling out the hook.

### Statistical analyses

A Student's unpaired *t*-test was used to compare the size of male and female sharks caught during the study, while a one-way analysis of variance (ANOVA) was used to compare the size of sharks among sampling locations. Murchie *et al.* (2010) showed that juvenile lemon sharks tagged in Page Creek, Kemp's Creek and Broad Creek exhibit a high degree of fidelity and move between these three tidal creeks, but rarely move to Poison Flat and Starved Creek. As such, for the purpose of the comparison among sites, we grouped sharks caught in Page, Kemp's and Broad Creeks and compared their total lengths with that of sharks caught in Poison Flat and Starved Creek (without site-specific differentiation). Regression analysis was used to quantify the relationship between total length and fight time (Zar, 1999). If the assumptions of a linear model could not be met, data were transformed accordingly.

A multivariate principal component analysis (PCA) was used to relate characteristics of the physiological response of sharks to elements of the angling event. The angling event was defined from the time of hook-up to time of release. A PCA with varimax rotation (Kaiser, 1960; Tabachnick and Fidell, 1989) was used to model the physiological parameters, including blood glucose and lactate, packed cell volume and plasma chloride, potassium, sodium, magnesium, calcium, BUN and creatinine. Total length and water temperature were also included in the analysis because they could covary with the physiological response. Principal factors with eigenvalues >1 were used to examine the relationship between physiological factors of fish and metrics of the angling event. Factors with eigenvectors >0.4 or <− 0.4 were used to characterize each principal component (Kaiser, 1960). These rotated principal components were regressed (simple linear regression) against fight time and time to bleed. We used time to bleed as an independent variable because the time course of the physiological stress response was likely to continue into the handling phase of the angling event (Cooke *et al.*, 2013). For principal components that were significantly related to fight time or time to bleed, we then used regression analysis to evaluate the relationship between individual physiological blood parameters that weighted these axes significantly to both fight time and time to bleed. Logistic regression was used to determine whether there was any relationship between rotated principal component scores and the incidence of post-release mortality.

The RAMP scores and short-term behaviour were compared qualitatively with the incidence of mortality post-release. Contingency analysis was used to determine whether there was any relationship between RAMP scores and the post-release mortality.

All analyses were performed using JMP 10.0.0 (SAS Institute, Cary, NC, USA), and the level of significance (α) for all tests was 0.05.

## Results

A total of 32 juvenile lemon sharks were captured, ranging in size from 530 to 875 mm total length (mean, 677 ± 81 mm). Only two of the captured sharks did not have an umbilical scar, indicating that the majority (94%) were neonates. An equal number of male and female juvenile lemon sharks were caught, and there was no significant difference in size between sexes (males, 655 ± 60 mm; females, 699 ± 86 mm; *t*-test, *P * < * *0.1). The total length of sharks differed significantly across sites (ANOVA, d.f. = 31, *F* = 7.47, *P* = 0.002), with sharks from Starved Creek (mean, 767 ± 57 mm total length, *n* = 6) being significantly larger than those from Poison Flat (mean, 680 ± 93 mm, *n* = 10) and from the Page, Kemp's and Broad Creeks (mean, 642 ± 49 mm, *n* = 16). The water temperature at the point of capture for all sharks ranged from 28.7 to 35.2°C (mean, 31.0 ± 1.7°C) and was significantly warmer at Starved Creek (33.9 ± 1.6°C) then at Poison Flat and Page, Kemp's and Broad Creeks (ANOVA, d.f. = 31, *F* = 11.37, *P* = 0.0002).

Fight time ranged from 43 to 476 s (mean, 193 ± 102 s) and was positively related to the total length of the shark (*r*^2^ = 0.25, d.f. = 31, *P* = 0.014). No sharks were preyed upon or died prior to being landed. Of the 32 sharks landed, four (12.5%) suffered mortality post-release; two at Kemp's Creek (610 and 660 mm total length) and two at Starved Creek (770 and 870 mm total length). The mean size of sharks that suffered mortality post-release (727 ± 116 mm) was not significantly different from the size of sharks that survived (670 ± 74 mm; Mann–Whitney *U*-test, *P* = 0.34; Table 1).

**Table 1.** Body size, capture metrics, physiological measurements and reflex action mortality predictor (RAMP) scores for juvenile lemon sharks following catch-and-release angling at Cape Eleuthera, The Bahamas

Measurement	No mortality (*n* = 28)		Mortality (*n* = 4)	
	Mean (SD)	Range	Mean (SD)	Range
Total length (mm)	670 (74)	530–875	727 (116)	610–870
Fight time (s)	194 (105)	43–476	186 (84)	107–295
Length-corrected hooking depth (proportion)	0.14 (0.05)	0.07–0.24	0.165 (0.002)	0.164–0.167
Glucose (mmol/l)	5.59 (1.33)	3.67–8.96	5.95 (1.96)	4.86–8.88
Lactate (mmol/l)	3.72 (1.37)	0.32–6.31	3.23 (0.84)	2.31–4.35
Packed cell volume (fraction)	0.21 (0.05)	0.09–0.33	0.19 (0.29)	0.18–0.24
Chloride (mmol/l)	271.8 (11.8)	240–290	282 (10.5)	269–293
Calcium (mequiv/l)	5.14 (0.6)	3.89–6.31	5.21 (0.61)	4.46–5.73
Magnesium (mmol/l)	3.46 (1.26)	2.68–9.39	3.56 (1.10)	2.84–5.21
Sodium (mmol/l)	277.2 (30.8)	149–327	280.7 (24.5)	253–301
Potassium (mmol/l)	7.67 (0.91)	6.02–9.81	7.41 (1.66)	6.02–9.81
Blood urea nitrogen (mmol/l)	48.9 (2.5)	42.6–53.5	50.1 (1.6)	48.4–51.9
Creatinine (mg/dl)	0.37 (0.15)	0–0.67	0.35 (0.06)	0.31–0.44
Blood urea nitrogen:creatinine (mmol/l)	136.3 (58.5)	72.3–368.0	144.6 (19.4)	115.9–157.3
Water temperature (**°**C)	31.3 (1.6)	28.7–35.2	33.2 (2.0)	31.4–35.1
RAMP score (proportion)	0.09 (0.2)	0–0.5	0.44 (0.3)	0–0.75

The parameter mean, one standard deviation and minimum and maximum values (range) are displayed for sharks that did and did not suffer mortality during the 15 min post-release observation period.

### Hooking injury

Hook depth ranged from 55 to 150 mm (mean, 100 ± 33 mm), and length-corrected hooking depth ranged from 0.07 to 0.24 (mean, 0.15 ± 0.04). The majority of hooking locations were considered non-critical, including the basihyal (*n* = 18) and the corner of the jaw (*n* = 9). Additional notes were taken when observing the hook placement in the basihyal, and there were an equal number of sharks hooked in the front vs. the back of the basihyal (*n* = 9 for each). Four sharks (12.5%) were hooked in critical locations, including the gut (*n* = 1), the eye (*n* = 1) and the cranial part of the oesophagus (*n* = 2), and none of these sharks died during the post-tracking period. The four sharks that suffered mortality had an almost identical length-corrected hooking depth (range, 0.164–0.167; Table 1), which corresponded to being hooked in the basihyal; one in the front of the basihyal and three in the back (mean length-corrected hooking depth of 0.165 ± 0.002).

A total of eight sharks (25%) experienced bleeding, but the incidence of blood was not related to hooking location, and no sharks hooked in critical locations bled. One shark that was hooked in the back of the basihyal did bleed excessively through the gill slits following hook removal, yet this shark did not die during the post-release tracking period.

### Physiological assessments

Principal component analysis performed with blood parameters produced four factors that described 66.5% of the variance in the variables used in this study (Table 2). Principal component 1 (PC1) accounted for 23.5% of the variance and was characterized by high positive factor loadings for blood lactate, packed cell volume, potassium and creatinine, low magnesium, BUN and BUN:creatinine ratio, and collectively represented a response predominantly related to exercise (Table 2). Principal components 2, 3 and 4 (PC2, PC3 and PC4) accounted for 15.7, 14.0 and 13.5% of the variance, respectively. Principal component 2 was characterized by high factor loading for chloride, magnesium, sodium and potassium, representing a response in blood ionic concentrations. Principal component 3 was characterized by high blood lactate and calcium, and low blood glucose and BUN (Table 2). Principal component 4 was characterized by high factor loading for water temperature and total length, and low loading for potassium (Table 2).

**Table 2.** Loading of blood physiology parameters for juvenile lemon sharks into four principal factors by principal component analysis

Measurement	PC1	PC2	PC3	PC4
Eigenvalue	3.64	2.47	1.30	1.23
Glucose	−0.004	0.030	−0.713*	−0.223
Lactate	0.630*	−0.173	0.539*	−0.149
Packed cell volume	0.412*	−0.653*	0.099	−0.074
Chloride	0.027	0.736*	−0.269	0.018
Calcium	−0.149	−0.219	0.745*	0.053
Magnesium	−0.537*	0.611*	0.311	−0.093
Sodium	−0.118	0.510*	−0.082	−0.218
Potassium	0.451*	0.546*	−0.140	−0.470*
Blood urea nitrogen (mmol/l)	−0.467*	0.128	−0.446*	−0.341
Creatinine	0.921*	−0.116	−0.117	−0.053
Blood urea nitrogen:creatinine	−0.890*	0.107	0.022	−0.138
Total length	0.230	−0.149	0.215	0.670*
Water temperature	−0.119	−0.008	0.047	0.901*
Percentage of variance explained	23.5	15.7	14.0	13.5

Variances and loading values are products of varimax factor rotation. *Variables that contributed maximally to each principal component are >0.4 or <− 0.4.

Of the principal components generated, only PC1 and PC3 were significantly related to fight time (PC1, *r*^2^ = 0.32, d.f. = 28, *P* = 0.001; and PC3, *r*^2^ = 0.19, d.f. = 28, *P* = 0.019) and time to bleed (PC1, *r*^2^ = 0.34, d.f. = 28, *P* = 0.0009; and PC3, *r*^2^ = 0.28, d.f. = 28, *P* = 0.003), with both scores increasing with duration. Of the physiological parameters that loaded significantly on PC1, only blood lactate (*r*^2^ = 0.28, d.f. = 31, *P* = 0.002), potassium (*r*^2^ = 0.22, d.f. = 32, *P* = 0.006) and creatinine (*r*^2^ = 0.14, d.f. = 31, *P* = 0.03) were positively related to fight time, while only blood lactate (*r*^2^ = 0.30, d.f. = 31, *P* = 0.001) and potassium (*r*^2^ = 0.12, d.f. = 31, *P* = 0.043) were positively related to time to bleed. For PC3, BUN was negatively related to time to bleed (*r*^2^ = 0.18, d.f. = 31, *P* = 0.01).

Of the four principal components, only PC4 was significantly related to post-release mortality during the tracking period (logistic regression, χ^2^ = 3.87, *P* = 0.049).

### Reflex indices and post-release tracking

A total of 22 sharks (68%) showed no sign of reflex impairment for any of the four indices used (Table 3). Of the 10 sharks that exhibited impaired reflexes, nine lost the BITE reflex, four did not elicit NM reflex, three did not respond to FLEX, and two lost EQUIL upon release. Of these 10 sharks, seven were hooked in the basihyal, two in the corner of the jaw, and one in the oesophagus. Of the nine sharks that lost the BITE reflex, six were hooked in the basihyal, four in the back of the basihyal, and two in the front. A total of five sharks showed reflex impairment for more than one trait; three sharks being impaired in two of the four indices, and two sharks being impaired in three of the four measures (RAMP = 0.75). Post-release mortality was positively related to RAMP (Pearson's χ^2^ = 9.56, *P* = 0.022). One of the sharks that died post-release was impaired in three of the four reflex indices (BITE + EQUIL + FLEX; RAMP = 0.75). Two of the sharks that died post-release were impaired in two of the reflex indices (BITE + NM and BITE + FLEX; RAMP = 0.5), while the remaining mortality showed no signs of reflex impairment based on the four indicators used.

**Table 3.** Impairment of individual reflexes [reflex action mortality predictors (RAMP) index]

RAMP impairment index (total no. per proportion)	BITE	NM	FLEX	EQUIL
0 (*n* = 22)	0	0	0	0
0.25 (*n* = 4)	0.75	0.25	0	0
0.50 (*n* = 4)	1.00	0.50	0.25	0.25
0.75 (*n* = 2)	1.00	0.50	0.50	1.00

Values represent the proportion of individuals with a particular reflex impaired within each group, with 0 = no impairment and 1 = all reflexes impaired. Abbreviations: BITE, bite response; EQUIL, equilibrium at release; FLEX, flex of the body; and NM, ability to close the nictitating membrane over the eye.

All sharks SD for a proportion of the tracking period (ranging from 10 to 100%; mean 83 ± 28%). A total of nine sharks SND during the tracking period, with seven of these sharks SND for <10% of the time, and the other two SND for 13 and 29%, respectively. None of the sharks that SND died post-release. A total of 14 sharks (34%) exhibited NM for a portion of the tracking period, but only after SD or SND (i.e. none of these sharks exhibited NM immediately following release). Five sharks that SND also exhibited NM at some point during the tracking period. All four sharks that suffered mortality showed NM for 76–90% of the tracking period. Eight of the sharks that exhibited NM did so for <10% of the tracking period, while the remaining two sharks were NM for 33 and 54%, respectively. The shark that exhibited NM for 54% of the time initially SD for 20 s following release, stopped for nearly 8 min, and then SD for the remainder of the tracking period. This was the only shark in the study that was gut hooked, and it was caught at the mouth of Starved Creek within an hour of two other sharks in the study that suffered mortality post-release at the same location.

## Discussion

The present study is one of only a few to date to have estimated the short-term delayed mortality of elasmobranchs following catch-and-release angling. We observed a 12.5% mortality rate for juvenile lemon sharks following catch-and-release angling. This rate is lower than the 26% mortality of common thresher sharks (*Alopias vulpinus*) following catch-and-release recreational angling off the California coast (Heberer *et al.*, 2010) and the 24% (72 h) mortality reported in hook-and-line caught dogfish (*Squalus acanthias*) in the Gulf of Maine (Mandelman and Farrington, 2007a), although pen effects could have artificially inflated mortality estimates in the latter study. Lemon shark mortality was also much lower when compared with the at-vessel mortality observed in carcharhinid sharks in commercial hook fisheries such, as pelagic longline [e.g. dusky, *Carcharhinus obscurus* (49%); silky, *Carcharhinus falciformis* (66%); and night, *Carcharhinus signatus* (81%); Beerkircher *et al.*, 2002] or demersal longline [sandbar, *Carcharhinus plumbeus* (36%); dusky (81%); and blacktip, *Carcharhinus limbatus* (88%); Morgan and Burgess, 2007]. The far higher mortality associated with these commercial longline gears is logical given that hook times normally extend to many hours, while catch-and-release angling hook times for small sharks are of the order of minutes. Comparatively, previous studies for recreationally angled teleosts have showed median mortality rates following the release of 11%, with considerable variation in mortality rates across species, angling techniques and gear type (reviewed by Bartholomew and Bohnsack, 2005). Mortality rates above 20% resulting from catch-and-release angling are typically considered to be unacceptable (Arlinghaus *et al.*, 2007). While not a trivial degree of mortality by recreational catch-and-release standards, the 12.5% accounted for in the present study suggests that juvenile lemon sharks are not immune to the impacts associated with catch-and-release angling. Given that our post-release monitoring period was relatively short (only 15 min), additional post-release mortalities could have occurred, thus making the mortality estimate in this study somewhat conservative.

Hooking damage is relatively common in sharks and can impact survival post-release (reviewed by Skomal, 2007). Although not considered a critical hooking location, all four sharks that died following release were hooked in the basihyal. In addition, 90% (nine of 10) sharks that showed impaired BITE reflex were also hooked in the basihyal. Given that lemon sharks are capable of buccal pumping (Compagno, 1984), hooking in the basihyal may have reduced the ability to move water over the gills during capture, following release, or both. A reduction dissolved oxygen levels related to higher water temperatures may have further impeded the ability of sharks to recover following release, hence the positive relationship between PC4 scores and mortality. Moreover, those sharks that died following release exhibited reduced swimming and frequently rested on the substrate, potentially because of difficulty in recovering from angling. Although circle hooks are intended to hook the catch in the corner of the jaw and other non-critical locations (Kerstetter and Graves, 2006; Afonso *et al.*, 2011; Cooke *et al.*, 2012), a nearly equal number of sharks were hooked in the basihyal and corner of the jaw (10 and nine, respectively). If circle hooks frequently hook juvenile lemon sharks in the basihyal, then other types and sizes of hooks, as well as different fishing techniques, might need to be considered during recreational angling for juvenile lemon sharks as a way to reduced hooking mortality. Further research is needed to decouple the specific impacts between hooking in the basihyal and respiration in juvenile lemon sharks, especially in different temperature/dissolved oxygen regimens.

The relatively short fight times and short post-release monitoring period in our study may have reduced our ability to detect any relationship between physiological disturbances and mortality of juvenile lemon sharks. The time course response of many physiological parameters measured in our study may not peak for hours after the stress event (reviewed by Cooke *et al.*, 2013). Brooks *et al.* (2011) conducted a time course study on juvenile lemon sharks when testing the impacts of tonic immobility. Although sharks in the study by Brooks *et al.* (2011) were not stressed via recreational angling, they showed that the disturbance associated with capture (for example, blood lactate levels) may not peak for 180 min following the onset of a stressor. This suggests that blood lactate and other blood physiological measurements in our study may under-represent the peak concentrations resulting from the exercise associated with recreational angling in this species.

Although short-term mortality was not related to blood physiology, the magnitude of physiological disturbance experienced by angled sharks was positively related to fight time and time to bleed. In our study, blood lactate loaded positively into both PC1 and PC3, and univariate analyses showed a positive relationship between fight time and blood lactate concentrations. This is consistent with previous findings that blood lactate levels associated with capture-related burst swimming and anaerobiosis is related to the duration of angling in sharks (Heberer *et al.*, 2010), as well as teleosts fishes (Cooke *et al.*, 2013). Of the other factors that loaded significantly on the principal component axes that showed a positive relationship with fight time and time to bleed, only potassium and creatinine, independently, were positively related to fight time, although these trends were relatively weak. Nevertheless, acute hyperkalaemia is commonly observed in physiologically stressed elasmobranchs (e.g. Cliff and Thurman, 1984; Manire *et al.*, 2001), with myocardial disruption suggested to ensue when plasma potassium concentrations exceed 7 mmol/l (Martini, 1974). Indeed, a clear association between moribund gummy sharks (*Mustelus antarcticus*) exhibiting muscle tetany and elevated K^+^ concentrations have been reported in the hours following fishing capture stress (Frick *et al.*, 2010). The range in potassium for our study was 6.0–9.8 mmol/l (mean, 7.65 ± 1.01 mmol/l), and there was no clear relationship in K^+^ concentrations between those sharks that died or survived (Table 1). Recovery from acutely elevated potassium has been previously reported in dogfish (Mandelman and Farrington, 2007b). In fact, none of the physiological parameters measured in our study differed between sharks that suffered mortality during the 15 min tracking period and those that did not. Our ability to relate the blood physiology to post-release mortality could have been confounded by relatively short angling times and a lack of physiological time course response values for juvenile lemon sharks caught via recreational angling. However, longer angling times may not be realistic for the size of sharks used in our study, and perhaps juvenile lemon sharks are relatively robust to the physiological stressors associated with recreational angling. Longer angling times typically experienced by larger sharks targeted by recreational anglers may indeed result in greater physiological stress, leading to higher post-release mortality, yet a comprehensive assessment of the size-dependent response of sharks to recreational angling remains to be carried out.

The measurement of reflex impairment could prove to be a good assessment of vitality and short-term post-release mortality in juvenile lemon sharks. In our study, mortality was higher for individuals with higher RAMP scores, potentially indicating cumulative impacts associated with hooking injury, physiological response to angling stress and higher water temperatures. Water temperatures in the present study averaged above 30°C, and elevated water temperature has been shown to exacerbate capture-related stress in sharks (Manire *et al.*, 2001). Based on our results, a simple way in which recreational anglers could help to reduce angling-related mortality of juvenile lemon sharks is to avoid capture at high water temperatures (>31°C). A larger sample size and a wider range of water temperatures, as well as tracking sharks for longer durations (∼1 h), may have provided further insights into the potential physical and physiological impacts of catch-and-release recreational angling on juvenile lemon sharks. Given the relatively high site fidelity of juvenile lemon sharks, this species and life stage could prove to be an excellent candidate for quantifying the impacts of repeat capture and sublethal effects of catch-and-release recreational angling. Additional work can also be done to examine the most effective way in which recreational anglers can assist in the recovery of juvenile lemon sharks prior to release.

Our study suggests that if juvenile lemon sharks are caught and released in benign conditions, recreational angling can be a popular activity with relatively low mortality rates. Nevertheless, given that sharks are important marine predators, it remains uncertain whether even low mortality rates from catch-and-release recreational angling can affect population-level traits, with cascading effects through marine ecosystems. Given that the incidence of catch-and-release angling of sharks is increasing, more studies on the physical and physiological impacts of recreational angling on a greater diversity of sharks and range of life-history stages are needed to help guide shark management and conservation measures (Gallagher *et al.*, 2012).
